# Assessment of Knowledge of Breast Cancer and Screening Methods among Nurses in University Hospitals in Addis Ababa, Ethiopia, 2011

**DOI:** 10.1155/2013/470981

**Published:** 2013-08-06

**Authors:** Semarya Berhe Lemlem, Worknish Sinishaw, Mignote Hailu, Mesfin Abebe, Alemseged Aregay

**Affiliations:** ^1^Department of Nursing, University of Mekele, Ethiopia; ^2^School of Nursing and Midwifery, Addis Ababa University, Ethiopia; ^3^Department of Nursing, University of Gondar, Ethiopia

## Abstract

*Background*. According to the American Cancer Society, about 1.3 million women will be diagnosed with breast cancer annually worldwide and about 465,000 will die from the disease. In Ethiopia breast cancer is the second most often occurring cancer among women. Early diagnosis is especially important for breast cancer because the disease responds best to treatment before it has spread. *Objective*. To assess knowledge of breast cancer and screening methods among nurses in university hospitals. *Method*. This cross-sectional descriptive study used simple random sampling on sample of 281 nurses. Structured questionnaires draw out responses about knowledge and screening method of nurses in regard to breast cancer. Bivariate analysis was used principally and variables were then entered to multiple logistic regressions model for controlling the possible effect of confounders and the variables which have significant association were identified on the basis of OR, with 95% CI and *P* value. *Results*. The findings of this study revealed that only 156 (57.8%) of them were knowledgeable about breast cancer and its screening and 114 (42.2%) were not. Knowledge of breast cancer was found to be significantly associated with regular course in nursing, family history of respondents, and unit of work. *Conclusion and Recommendation*. The results of this study indicate that the knowledge of nurses is not satisfying and highlight the need to improve the content in the nursing curriculum and to undergo more workplace training in the area of breast cancer and screening methods.

## 1. Background

Globally, cancer is one of the top ten leading causes of death. It is estimated that 7.4 million people died of cancer in 2004 and, if current trends continue, 83.2 million more will have died by 2015. Among women, breast cancer is the most common cause of cancer mortality, accounting for 16% of cancer deaths in adult women [1]. 

Breast cancer is a major life-threatening public health problem of great concern. Long-term increases in the incidence of the disease are being observed in both industrialized and developing world [2].

Data from South Africa's National Cancer Registry (NCR) show breast cancer as the leading cancer among women. South African women have a 1 in 29 lifetime risk of developing breast cancer, with an age-standardized incidence rate of 30.6 per 100,000 populations. These rates vary by race group, with Black women having the lowest (16.3) and White women the highest (69.4) rates of breast cancer diagnosis [3]. 

Breast cancer is the third commonest cancer in women in Uganda after Kaposi's sarcoma and cervical cancer. Breast cancer incidence in Uganda is 22: 100,000. Five-year survival rate is 56%. In Nigeria, the incidence of breast cancer has been reported to be 33.6/100,000 [4, 5].

Breast cancer is the second most often occurring cancer (cervical cancer is first) among women in Ethiopia. It is estimated that around 10,000 Ethiopian women and men have breast cancer with thousands of more cases unreported as women living in rural areas often seek treatment from traditional healers before seeking help from the government health system [6].

During 1995–99, 137 biopsy proven breast cancer cases underwent surgical treatment at Tikur Anbessa Hospital, Addis Ababa. Of these cases, records of 125 were retrieved and analyzed to assess the pattern and treatment outcome of the disease. The median age of females was 40 years. The median duration of the presenting symptom on admission was nearly 1 year. Clinically, the majority of cases had stage III disease. Invasive ductal carcinoma was the most frequent type. Eighty-nine (71.2%) patients underwent modified radical mastectomy. During a short followup, 50 (45.9%) of 109 patients were seen with recurrences. Only 4 cases were seen at 5 or more years [2].

A prospective study conducted at Tikur Anbessa was designed to obtain information on demographic characteristics, clinical profile, and problems related to early diagnosis and treatment of breast cancer in 72 (62 females and 10 males) Ethiopian patients, the female to male ratio being 6.2 : 1, and the females in this series developed breast cancer at a younger age (72% were premenopausal) and 76% had advanced disease (stages III and IV) at presentation [7].

Education about the importance of early detection in decreasing mortality rates might be of value in raising awareness of the various methods of early detection of breast cancer. More research is also needed to identify the underlying variables that might influence nurses' own practice of early detection methods of breast cancer. Empowering nurses with information about early detection methods and their related benefits could help in advancing their skills in performing breast self-examination and expanding their role as client educators [4].

Looking at the higher figure of breast cancer globally at current and future, great emphasis must be given to the issue of breast cancer and its screening to reduce the mortality rate. So far, no data has been found in Ethiopia that revolves on assessing nurses' knowledge and screening of breast cancer. As a result, the outcomes of this study will 7 serve as a baseline data for further findings.

According to the result obtained from this study, if nurses' knowledge towards breast cancer and its screening is remarkable, it lets health policy makers strengthen the trend being taken, but if the reverse occurs the finding will serve as a source for health policy planners to design strategy that can reshape and fill the gap.

In addition the result of this study will have direct implication to the growth of nursing profession towards early detection and prevention of complication. Furthermore to appreciate the nurse's stand to this world warning issue and to equip them based on the identified gaps as to that they can be able to manage problems of the community they serve.

Several studies have reported that breast cancer is the most common cancer and principal cause of cancer deaths in women and is therefore a world concern. 

## 2. Methods

### 2.1. Study Setting

The study was carried out in Addis Ababa which is the Federal Capital of Ethiopia. The city has 37 health posts, 15 health stations (8 private and 7 governmental), 29 health centers (24 owned by MOH and 5 by others), and grand total of 30 hospitals. Out of these hospitals some are under Federal Ministry of Health and other by Addis Ababa Regional Bureau. Among these, Black Lion Hospital and St. Paul General Specialized Hospital are under the Federal Ministry of Health, are integrated with Addis Ababa University, and are registered as university hospitals [8].

### 2.2. Study Design, Participants, and Sampling Procedure

A descriptive cross-sectional study design was used and study participants were nurses working at university hospitals of Addis Ababa. Sample size was determined using the formula for single population proportion with a 95% confidence interval, a precision of 5%, and an assumed prevalence of cervical cancer 50% (0.5) to get a maximum sample size as there was no previous study conducted similar to this study. The calculated sample size was 256 and by adding 10% nonresponse rate and incomplete lost questionnaire, the total sample size was found to be 281 nurses. The study subject again was selected proportionally to size allocation to give equal chance to each teaching hospital and then simple random sampling technique was utilized. Data was collected using a pretested structured self-administered questionnaire.

### 2.3. Data Management and Analysis

Data entry and validation were done in EPI info version 3.5.1 statistical software data which was then exported to SPSS windows version 16.0 where frequencies and statistical analyses were run. The outcome variables of the study (knowledge and screening methods) were binary categorical variables (knowledgeable and not knowledgeable). The median value was used to categorize nurses as knowledgeable or not knowledgeable about breast. 

Accordingly, the sum value less than the median was categorized as not knowledgeable and the value greater than or equal to the median was categorized as knowledgeable. Bivariate analysis was used primarily to check which variables have association with the dependent variable individually. 

Variables found to have association with the dependent variables were then entered into multiple logistic regression model for controlling the possible effect of confounders and finally the variables which have significant association were identified on the basis of OR, with 95% CI and *P* value.

### 2.4. Ethical Consideration

The study sought ethical approval from Addis Ababa University College of Health Sciences Department of Nursing and Midwifery research review committee. Written informed consent was obtained from the respondents, clearly stating potential risks and benefits of the study and sought their voluntary participation. 

## 3. Results

### 3.1. Response Coverage

Two hundred seventy nurses out of 281 eligible subjects completed and returned the questionnaires, giving a response rate of 96%. The majorities 180 (66.7%) of the respondents were from Black Lion hospital.

### 3.2. Sociodemographic Characteristics of Respondents

Of those nurses who responded (270) giving a response rate of 96% out of this 171 (63.3%) were females and 99 (36.7%) male. Age range of participants was from 21 to 58 years (mean = 29.8; SD = 8.14) and as to the marital status 169 (62.6%) were single and 87 (32.2%) married. The nurses who participated were from medical ward 107 (39.6%), surgical ward 54 (20.0%), labor 9 (3.3%), gynecology 47 (17.4%), oncology 13 (4.8%), and others 39 (14.4%) (Table 1).

### 3.3. History of Breast Cancer among Study Participants

Of the respondents 165 (61.1%) had clinical experience of 5 years and less. The majority of the participants had no history of breast disease and family history of breast cancer as follows: 248 (91.9%), 213 (78.9%), respectively. Of the respondents 149 (55.2%) had looked after patient with breast cancer and the remaining 121 (44.8%) never nursed (Table 2).

### 3.4. Knowledge of Breast Cancer

**Figure 1:**
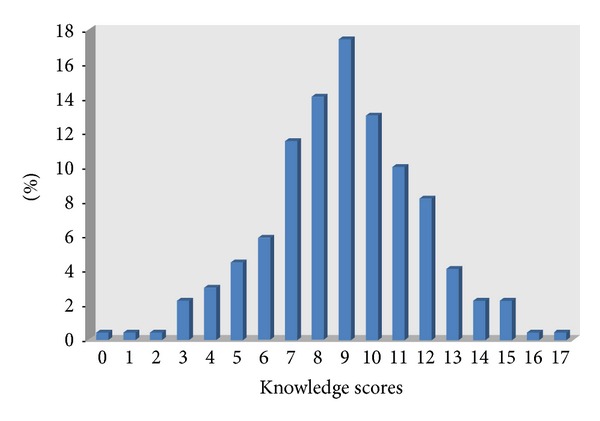
Distribution of knowledge scores of study participants at the university hospitals of Addis Ababa, Ethiopia, March 2011. *N* = 270, median = 9.

Distribution of knowledge scores on breast cancer and screening methods amongst nurses in the university hospital ranges from 0 to 17, or 0%−100% correct. The mean score on the knowledge test was 8.9, SD = 2.8, and the median score was nine. And out of the 270 nurses 57.8% the nurses scored ≥9 (Figure 1). 

### 3.5. Source of Information for Breast Cancer

**Figure 2:**
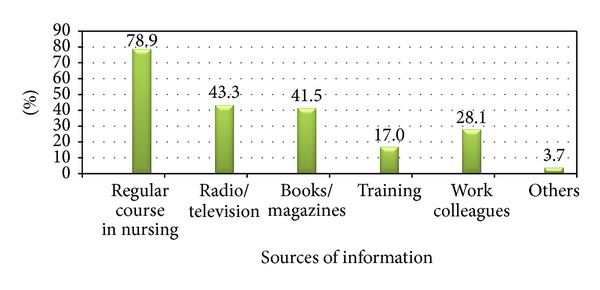
Source of information about breast cancer among nurses in university hospitals of Addis Ababa, Ethiopia, March 2011. Percent may exceed 100% as multiple answers are possible.

As a major source of information for breast cancer mentioned by the 270 nurses who participated in the study was regular course in nursing 213 (78.9%) and training 46 (17%), 41.5% and 43.3% were from radio and television and reading books, respectively, and very few of them from other sources (3.7%) (Figure 2).

### 3.6. Knowledge about Risk Factors of Breast Cancer

Regarding the knowledge of risk factors for breast cancer, 241 (89.3%) knew that there are risk factors involved in the development of breast cancer, while 24 (8.9%) said no and 4 (1.5 %) claimed they do not know. About 81 (30.0%) of those who knew that there is a risk factor were able to mention two and more correct risk factors. From these more than half of the respondents, 188 (69.6%), mentioned family history of breast cancer as a risk factor and other risk factors like wearing tight bra 53 (19.6%), prolonged breast feeding 27 (10%), and multiparity 38 (14.1 %) cracked nipple as a risk factor.

### 3.7. Knowledge of Nurses about Signs of Breast Cancer

**Figure 3:**
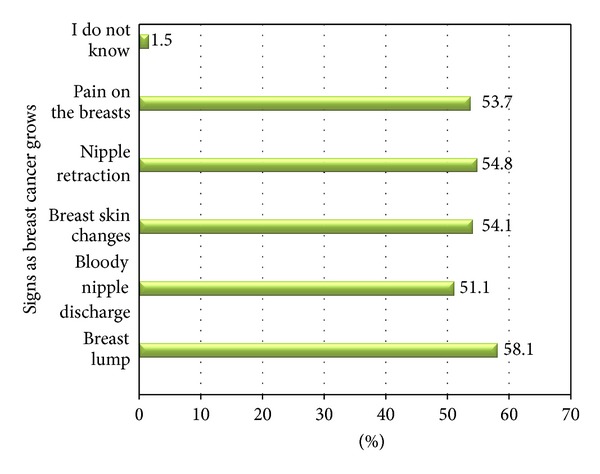
Distribution of study subjects by their frequency of knowing signs of breast cancer, at university hospitals of Addis Ababa, March 2011. Percent may exceed 100% as multiple answers are possible.

Concerning early breast cancer 122 (45.2%) respondents mainly mentioned that it does not cause pain and symptom. Regarding the stage of breast cancer curability 199 (73.7%) respond at stage of 0 and I followed by 55 (20.4%) at stage 0, I and II and 15 (5.6%) agreed it is not curable at all. As shown in figure three regarding signs as breast cancer grows, breast lump was the predominantly mentioned symptom by the respondents 157 (58.1%) followed by nipple retraction, breast pain, breast skin change, bloody nipple discharge, and the remaining claimed they do not know (Figure 3).

### 3.8. Knowledge of Nurses about Screening Methods

A sequence of questions regarding screening of breast cancer was asked to assess the respondents' knowledge of breast cancer. Respondents were asked to state the early detection measures for breast cancer; 202 (74.8%) mentioned BSE, 120 (44.4%) identified breast examination by a health professional (CBE), and only 104 (38.5%) stated mammography as an early detection measures. Majority of the respondents 156 (57.8%) knew that breast cancer can be prevented at early stage of the disease; 89 (33.0%) of them responded it can be prevented before the disease manifests, while 19 (7.0%) said it cannot be prevented at all. Concerning treatment options for the disease 150 (55.6%) identified surgery; 95 (35.2%) and 112 (41.5%) knew that breast cancer can be treated with radiation therapy and chemotherapy, respectively. In addition one-fourth of the nurses 121 (44.8%) did respond that breast cancer can be successfully treated without mastectomy.

About 178 (65.9%) were aware that doing regular breast cancer screening has a great deal in curing breast cancer while 20 (7.4%) indicated it has little or no difference. 

For the question how often should breast self examination be performed, 139 (51.5%) of the study subjects reported that monthly 1–7 days after menses, together with that 194 (71.9%) respondents identified starting age to perform BSE to be at year of 20 (Table 3).

Knowledge of specific aspects of mammography revealed that age group most appropriate to start mammography screening and time interval 129 (47.8%) stated that 20 years every 2-3 years, 66 (24.4%) 30 year every 5 years and 75 (27.8%) 40 years every 1-2 year. 11 (4.1%) of the study participants reported that screening for mammography should be done weekly, 36 (13.3%) monthly, 134 (49.6%) every six months, and 89 (33%) yearly. 

**Figure 4:**
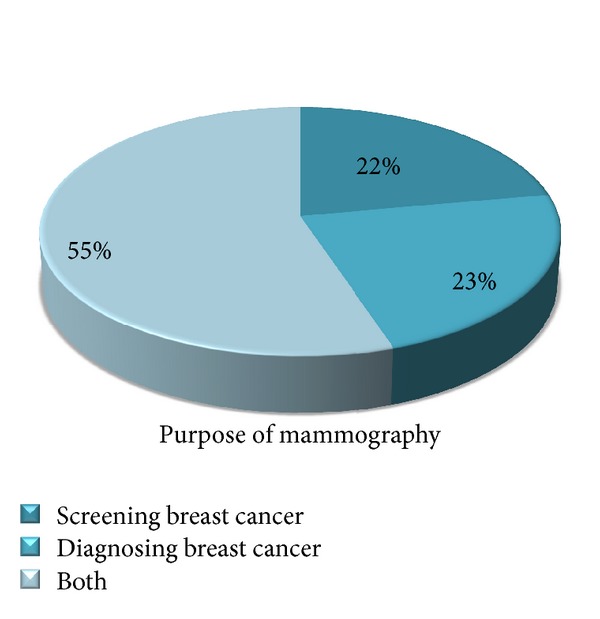
Distribution of study subjects by their knowledge of use of mammography, at university hospitals of Addis Ababa, March 2011.

Concerning the purpose 61 (22.6%) of the respondents were aware of mammography as a breast cancer diagnostic method rather than as a screening method (Figure 4). Median score was computed among the 270 respondents and 156 (57.8%) of them were knowledgeable about breast cancer and 114 (42.2%) were not knowledgeable with a score of median and above median.

### 3.9. Association of Sociodemographic Variable with the Knowledge of Nurses about Breast Cancer and Screening Method

Knowledge of breast cancer was found to be significantly associated with regular course in nursing (AOR = 3.874, 95% CI (1.908–7.866), *P* < 0.01). Also knowledge of breast cancer appears to be significant with family history of respondents. Nurses with family history of breast cancer were more likely to be knowledgeable than nurses with no family history of breast cancer (AOR = 3.042, 95% CI (1.636–5.656), *P* < 0.01). In addition knowledge of breast cancer was found to be significantly associated with the respondents unit of work. Nurses working in the oncology unit were (AOR = 4.865, 95% CI (1.895–26.439), *P* = 0.03) more knowledgeable than those working in surgical unit. Further significant association has been made with years of nursing experience. That is nurses working for 6–10 years were less likely to be knowledgeable (COR = 0.411, 95% CI (0.200–0.844), *P* = 0.015) than the nurses with nursing experience ≤5 years. Marital status also has a significant association with the knowledge of breast cancer in the aspect that unmarried respondents are (COR = 0.527, 95% CI (0.314–0.884), *P* = 0.015) more knowledgeable than married ones (Table 4).

Other sociodemographic factors like age, sex, history of breast disease, ever nursed patient with breast cancer, and nursing qualification were not found to be significantly associated with knowledge of breast cancer though age was significantly associated during analysis with simple logistic regression.

## 4. Discussion

The sociodemographic structure of this study identified that most of the respondents (38.9%) are between ages of 20 and 25 years; as the age increases the number of respondents decreases to the minimum value 7.4% of above 46 years. 62.6% and 32.2 were single and married, respectively, and greater than 60 of the participants were females with the remaining 36.7% being males. Moreover 49.3% had diploma in nursing; 50.7% are degree holders. Regarding the working experience 61.1% of them had five-year and less experience, 6.7% of them had 11–20 years, and 1.5% 31 had more years of work experiences. 39.6% and 20.0% of the nurses were working in medical and surgical units, respectively, and only 17.4 percent are assigned in gynecology unit. At last 8.9 percent of subjects had history of breast cancer and 21.1 in their family (Tables 1 and 2). 

The investigation has come up with findings like major source of information for breast cancer for the 270 nurses participated in the study, 78.9% to be regular course in nursing followed by radio/television 43.3%, 41.5% books, 28.1% work colleagues, 17% from training, and 3.7% of them mentioned internet as means of information which is consistent with a study conducted in 2004 in Singapore to assess breast cancer knowledge among healthcare professionals. The majority of the respondents 74% received breast cancer information via formal teaching both in school and in the workplace. Posters and brochures were the next frequently used portals of information, 42%. Other means included the television 24% and internet 19% [9]. The rationale for the slight difference could be due to economical factor and inadequate electronic media. 

### 4.1. Knowledge about Risk Factors of Breast Cancer

About 81 (30.0%) of those who knew there is a risk factor were able to mention four and more correct risk factors. From these it was found that family history of breast cancer 69.6%, smoking 54.4% and use of oral contraceptive 36.7%, increasing age 31.9%, and infertility 28.1% were well-distinguished risk factors. On the other hand, a small percentage of the nurses assumed that early menarche 24.4% and late menopause 18.9% were the risk factors of the breast cancer. In comparison with a cross-sectional study conducted among 125 nurses working in Pamukkale University Hospital in Denizli, of the nurses, 74 (57.6%) correctly knew at least four risk factors. It was found that increasing age 72%, familial history 94.4%, childlessness 85.6%, absence of breast feeding 82.4%, and taking birth control pill or hormone replacement therapy 50.4% were well-known risk factors. However, a small percentage of the nurses believed that early menarche 23.2% and late menopause 28.8% were the risk factors of the breast cancer [10]. The discrepancy may be due to; breast cancer being considered as disease of the developed countries much awareness and emphasis had been given to this issue in which one way or another making the nurses of Turkey be knowledgeable than nurses of university hospitals of Addis Ababa.

Results of this study also showed that 29.3% of the nurses were aware that obesity is a risk factor for breast cancer which is consistent with a cross-sectional survey conducted in seven teaching hospitals of Karachi, Pakistan, in 2003 which showed that 28% of the nurses knew that in some women being overweight increases the risk of developing breast cancer [11]. The similarity could be because of the fact that obesity is predisposing factor for most of chronic diseases.

### 4.2. Knowledge about Signs and Symptoms

On this study regarding signs as breast cancer grows, breast lump was the predominantly mentioned symptom by the respondents, 157 (58.1%), followed by nipple retraction, breast pain, breast skin change, and bloody nipple discharge. An enormous difference had been seen when compared with a descriptive study conducted on breast self-examination among nurses and midwives, in 2004 at the State Hospital, all public Health Cabins and Family Health Centers in the rural area of Izmir, western region of Turkey, as 70% of the subjects believed that the presence of masses (breast lumps) and nipple discharge were signs of breast cancer [12]. In 2004, a similar study was conducted in Singapore to assess breast cancer knowledge among healthcare professionals; the two most frequent symptoms named were a palpable breast lump and nipple discharge [10]. As it has been mentioned earlier the reason could be the disease being dominant in the developed countries and nurses working in these countries will be much more aware beside the theoretical course as they could practice it as well be able to differentiate signs of breast cancer.

### 4.3. Knowledge of Nurses on the Screening Method

When nurses were asked about BSE, 139 (51.5%) believed that it should be done monthly 1–7 days after menses and 202 (74.8%) of the study subjects were aware of BSE as an early detection measure for breast cancer. Of the two hundred seventy nurses only 89 (33.0%) said that screening for mammography should be done yearly. A cross-sectional study was conducted in Pamukkale University Hospital in Denizli; about BSE, 113 (90.4%) believed that it should be done monthly, and 85 (68%) believed that it should be done at luteal phase of menstruation [13]. Other cross-sectional descriptive study carried out to assess knowledge, attitudes and practice of breast cancer screening among female health workers in the two major government health institutions in Benin City, Edo State capital in Nigeria relatively low knowledge (45.5%) about Breast Self Examination (BSE) as a screening method was found [12].

 This gap might exist with the reason that the nurses in the western's are engaged in treating patient with breast cancer which makes them be alarmed about the frequency of performing BSE and its significance as early detection measure for disease of breast cancer. 

This study on knowledge of specific aspects of mammography revealed that only 75 (27.8%) 40 years every 1-2 year knew age group most appropriate to start mammography screening and only 89 (33%) of the study participants reported that screening for mammography should be done yearly. Concerning the purpose 61 (22.6%) of the respondents were aware of mammography as a breast cancer diagnostic method and 60 (22.2%) as a screening method, and the remaining percent knew MMG serves for both purposes. As to the result obtained from a cross-sectional descriptive study carried out in Nigeria the awareness of mammography as a diagnostic method was very high (80.7%), but an extremely low knowledge of mammography as a screening method was found [12]. And that of Pamukkale reported that all of the nurses who participated said that MMG should be done yearly [13]. Possible reason could be sufficient availability of mammogram which has its own impact on knowing the frequency to do mammography to nurses in the developed countries than nurses in developing countries.

### 4.4. Knowledge of Treatment Options

From this study concerning treatment options for the disease 150 (55.6%) identified surgery; 95 (35.2%) and 112 (41.5%) knew that breast cancer can be treated with radiation therapy and chemotherapy, respectively. In addition one-fourth of the nurses 53 (19.6%) did respond that breast cancer cannot be successfully treated without mastectomy. The results were congruent with a study conducted in 2004 in Singapore to assess breast cancer knowledge among healthcare professionals. 

Where treatment for breast cancer was concerned, 20% thought that a mastectomy was the available treatment. The majority (93%) was aware that, apart from surgery, other modalities such as radiotherapy and chemotherapy might be necessary [9].

This study regard to the overall knowledge of breast cancer, the median score was computed. Among the 270 respondents, 156 (57.8%) of them were knowledgeable about breast cancer and 114 (42.2%) were not knowledgeable with a score of median ≥9. This result goes in line with a study done in Singapore on knowledge and practice of breast cancer screening amongst public health nurses to assess their knowledge and practice of breast cancer screening. Response rate was 96.4%. Median knowledge score was nine and 58.3% of nurses scored >9 [14].

From this investigation knowledge of breast cancer was found to be significantly associated with regular course in nursing, family history of respondents, unit of work, years of nursing experience, and marital status. Other sociodemographic factors like age, sex, history of breast disease, ever nursed patient with breast cancer, and nursing qualification were not found to be significantly associated with knowledge of breast cancer. Some difference has been observed with a study that was carried out in Singapore; statistically significant factors influencing knowledge scores were related to the nursing profession, namely, nursing qualifications and current workplace. There was no significant association between knowledge score and age of the nurses, number of years in nursing, history of breast disease, or family history of cancer [12]. And other cross-sectional surveys conducted in seven teaching hospitals of Karachi, the largest city of Pakistan, in 2003 showed that graduates from private nursing schools, nurses who had cared for breast cancer patients, those having received a breast examination themselves, or those who never examined a patient's breast were more likely to have good knowledge [11].

## 5. Conclusion

In conclusion nearly half, 114 (42.2%), of the nurses working at the university hospital were not knowledgeable about breast cancer and its screening methods. It is possible to recommend a need to improve breast cancer content in the nursing curriculum; as the implementation of the revised curriculum may take some time, nurses, who are frontline medical professionals, should undergo more workplace training which will have an impact on narrowing the gap to make all nurses knowledgeable. The media should let a wide range of air time to provide comprehensive information about breast cancer as it can reach many nurses and at the same time many people. 

## Figures and Tables

**Table 1:** Frequency distribution of sociodemographic characteristics of respondents at the university hospital of Addis Ababa, Ethiopia, March 2011.

Variables	Frequency	Percent (%)
Age in years		
20–25	105	38.9
26–30	84	31.1
31–35	24	8.9
36–40	23	8.5
41–45	14	5.2
≥46	20	7.4
Sex		
Male	99	36.7
Female	171	63.3
Marital status		
Single	169	62.6
Married	87	32.2
Divorced	6	2.2
Widowed	7	2.6
Separated	1	4
Nursing qualifications		
Diploma	133	49.3
Degree	137	50.7
Nursing experience		
≤5	165	61.1
6–10	40	14.8
11–15	18	6.7
16–20	18	6.7
21–25	17	6.3
26–30	8	3.0
≥31	4	1.5
Current unit of work		
Medical ward	107	39.6
Surgical ward	54	20.0
Labor	9	3.3
Gynecology	47	17.4
Oncology	13	4.8
Others	39	14.4

**Table 2:** History of breast cancer among study participants at the university hospitals of Addis Ababa, Ethiopia, March 2011.

Variables	Frequency	Percent (%)
History of breast disease		
Yes	22	8.1
No	248	91.9
Family history of breast cancer		
Yes	57	21.1
No	213	78.9
Ever nursed a patient with breast cancer		
Yes	149	55.2
No	121	44.8

**Table 3:** Study participant's knowledge about BSE, at the university hospitals of Addis Ababa, Ethiopia, March 2011.

Variables	Frequency	Percent
Age to begin BSE		
20 years	194	71.9
25 years	40	14.8
30 years	12	4.4
35 years	24	8.9
How often should BSE be performed		
Every six months to time of ovulation	57	21.1
Monthly 1–7 days after menses	139	51.5
Once a week before rising out of bed	74	27.4

**Table 4:** Sociodemographic correlates of breast cancer knowledge of nurses in the university hospitals of Addis Ababa, Ethiopia, March 2011.

Variables	Knowledge of breast cancer, *N* = 270		Crude OR (95% CI)	Adjusted OR (95% CI)	*P* value
	Yes (≥9)	No (≤8)			
Age					
20–25	70 (25.9%)	35 (13.0%)	1.00		
26–30	44 (16.3%)	41 (15.2%)	0.537 (0.298–0.966)	0.947 (0.226–3.969)	0.94
31–35	14 (5.2%)	10 (3.7%)	0.700 (0.283–1.734)		
36–40	12 (4.4%)	11 (4.1%)	0.545 (0.219–1.359)		
41–45	8 (3.0%)	6 (2.2%)	0.667 (0.215–2.071)		
≥46	8 (3.0%)	11 (4.1%)	0.364 (0.134–0.986)	1.152 (0.196–6.770)	0.876
Sex					
Male	61 (22.6%)	38 (14.1%)	1.284 (0.775–2.128)	**	0.332
Female	95 (35.2%)	76 (28.1%)	1.00		
Marital status					
Unmarried	115 (42.0%)	68 (25.2%)	1.00		
Married	41 (15.2%)	46 (17.0%)	0.527 (0.314–0.884)	0.573 (0.313–1.049)	0.071
Nursing qualification					
Diploma	71 (26.3%)	62 (23.0%)	0.701 (0.431–1.138)	**	0.150
Degree	85 (31.5%)	52 (19.3%)	1.00		
Nursing experience					
0–5	141 (52.2%)	91 (33.7%)	1.00		
6–15	14 (5.2%)	22 (8.1%)	0.411 (0.200–0.844)	0.317 (0.141–0.714)	0.06
16–25	1 (0.4%)	1 (0.4%)	0.645 (0.040–10.448)		
Current unit of work					
Surgical	29 (10.7%)	25 (9.3%)	1.00		
Labor	8 (2.96%)	1 (0.4%)	6.897 (0.806–59.00)		
Medical ward	58 (21.48%)	49 (18.1%)	1.020 (0.529–1.967)		
Gynecology	26 (9.6%)	21 (7.8%)	1.067 (0.487–2.341)		
Oncology	11 (4.07%)	2 (0.7%)	4.741 (0.958–23.45)	4.865 (1.895–26.43)*	0.03
Regular course in nursing					
Yes	134 (49.6%)	79 (29.3%)	2.699 (1.479–4.924)	3.874 (1.908–7.86) *	0.00
No	22 (8.1%)	35 (13.0%)	1.00		
Training					
Yes	24 (8.9%)	22 (8.1%)	1.315 (0.696–2.486)		0.399
No	132 (48.9%)	92 (34.1)	1.00	**	
History of breast disease					
Yes	14 (5.2%)	8 (3.0%)	1.306 (0.529–3.227)	**	0.562
No	142 (52.6%)	106 (39.3%)	1.00		
Family history of breast disease					
Yes	24 (8.9%)	33 (12.2%)	0.446 (0.246–0.808)	3.042 (1.636–5.656)*	0.00
No	132 (48.9%)	81 (30.0%)	1.00		
Ever nursed a patient with breast cancer				**	0.132
Yes	80 (29.6%)	69 (25.6%)	0.686 (0.421–1.120)		
No	76 (28.1%)	45 (16.7%)	1.00		

-Average knowledge score ≥ 9.

*Statistically significant.

**Insignificant variables in the crude analysis were omitted from the multivariate analysis.

## Abbreviations

AAU: Addis Ababa University
BSE: Breast self-examination
B.L.H: Black Lion Hospital
CBE: Clinical breast examination
CHSDNM: College of Health Sciences Department of Nursing and Midwifery
ECA: Ethiopian Cancer Association
ENA: Ethiopian Nurses Association
FMOH: Federal Ministry of Health
MMG: Mammography
MOH: Ministry of Health.
